# Total contact cast wall load in patients with a plantar forefoot ulcer and diabetes

**DOI:** 10.1186/s13047-015-0119-0

**Published:** 2016-01-07

**Authors:** Lindy Begg, Patrick McLaughlin, Mauro Vicaretti, John Fletcher, Joshua Burns

**Affiliations:** Foot Wound Clinic, Department of Surgery, Westmead Hospital, Sydney, 2145 NSW Australia; Department of Surgery, Univeristy of Sydney, Westmead Hospital, Sydney, Australia; Centre for Chronic Disease Prevention, College of Health and Biomedicine, Victoria University, Melbourne, Australia; Institute of Sport, Exercise and Active Living, Victoria University, Melbourne, Victoria Australia; Arthritis and Musculoskeletal Research Group, Faculty of Health Sciences, The University of Sydney, Sydney, New South Wales Australia; Paediatric Gait Analysis Service of New South Wales, Sydney Children’s Hospitals Network (Randwick and Westmead), Sydney, New South Wales Australia

**Keywords:** Diabetes, Foot ulcer, Wound, Total contact cast, Offloading, Plantar pressure

## Abstract

**Background:**

The total contact cast (TCC) is an effective intervention to reduce plantar pressure in patients with diabetes and a plantar forefoot ulcer. The walls of the TCC have been indirectly shown to bear approximately 30 % of the plantar load. A new direct method to measure inside the TCC walls with capacitance sensors has shown that the anterodistal and posterolateral-distal regions of the lower leg bear the highest load. The objective of this study was to directly measure these two regions in patients with Diabetes and a plantar forefoot ulcer to further understand the mechanism of pressure reduction in the TCC.

**Methods:**

A TCC was applied to 17 patients with Diabetes and a plantar forefoot ulcer. TCC wall load (contact area, peak pressure and max force) at the anterodistal and posterolateral-distal regions of the lower leg were evaluated with two capacitance sensor strips measuring 90 cm^2^ (pliance®, novel GmbH, Germany). Plantar load (contact area, peak pressure and max force) was measured with a capacitance sensor insole (pedar®, novel GmbH, Germany) placed inside the TCC. Both pedar® and pliance® collected data simultaneously at a sampling rate of 50Hz synchronised to heel strike. The magnitude of TCC wall load as a proportion of plantar load was calculated. The TCC walls were then removed to determine the differences in plantar loading between the TCC and the cut down shoe-cast for the whole foot, rearfoot, midfoot and forefoot (region of interest).

**Results:**

TCC wall load was substantial. The anterodistal lower leg recorded 48 % and the posterolateral-distal lower leg recorded 34 % of plantar contact area. The anterodistal lower leg recorded 28 % and the posterolateral-distal lower leg recorded 12 % of plantar peak pressure. The anterodistal lower leg recorded 12 % and the posterolateral-distal lower leg recorded 4 % of plantar max force. There were significant differences in plantar load between the TCC and the cut down shoe-cast for the whole foot, rearfoot, midfoot and forefoot (region of ulcer). Contact area significantly increased by 5 % beneath the whole foot, 8 % at the midfoot and 6 % at the forefoot in the shoe-cast (*p* < 0.05). Peak pressure significantly increased by 8 % beneath the midfoot and 13 % at the forefoot in the shoe-cast (*p* < 0.05). Max force significantly increased 6 % beneath the midfoot in the (shoe-cast *p* < 0.05).

**Conclusion:**

In patients with diabetes and a plantar forefoot ulcer, the walls of the TCC bear considerable load. Reduced plantar contact area in the TCC compared to the shoe-cast suggests that the foot is suspended by the considerable load bearing capacity of the walls of the TCC which contributes mechanically to the pressure reduction and redistribution properties of the TCC.

## Background

Plantar neuropathic ulcers usually develop at sites of moderate to high repetitive cumulative load during normal walking [1–4]. It is recognised that plantar shear and shear-time integral magnitudes are also contributory factors [5]. Facilitation of healing occurs by reducing repetitive load to the ulcer [4]. Reducing plantar load at the site of an ulcer that is complicated by peripheral neuropathy continues to be a major challenge for clinicians and is critical to the outcome of care. If repetitive load persists, there is a chronic and ongoing disruption in the phases of healing producing cellular injury; which is recognised as one of the factors responsible for poor wound healing [4, 6–9]. It has been demonstrated histologically, that the application of a total contact cast results in the chronic ulcer resembling an acute wound in the reparative phase; that there is a reduction of inflammatory and reactive components, and an acceleration of reparative processes of the wound [10]. Since a neuropathic ulcer is a common precursor for amputation, an effective intervention should have a substantial impact on the prevention of amputation [11]. A fine balance must be achieved whereby plantar load is reduced to allow ulcer healing whilst allowing the patient to remain ambulatory. This is problematic since the thresholds for developing ulceration, likewise for healing a plantar ulcer, are yet to be established [3, 12–14].

The TCC is an effective intervention to reduce plantar pressure in patients with a neuropathic foot ulcer [15–18]. Offloading plantar neuropathic ulcers using a TCC is regarded as the ‘gold standard’ treatment for this condition [19–22] although the evidence supporting this has been referred to being of only moderate quality [17, 18, 23]. A TCC is generally regarded as a well-moulded, minimally padded cast that maintains contact with the entire plantar aspect of the foot and lower leg [1, 24–27] while providing protection of the wound from further injury [28, 29]. The TCC is traditionally non-removable and has been demonstrated to have superior wound healing outcomes compared to removable devices [17, 18, 25, 30]. This has been attributed to the “forced compliance” aspect of the design of the TCC [4, 19, 25, 31].

Over time, the TCC has undergone several modifications; Sifoam at the plantar metatarsal area [13, 26, 32], creation of a window at the site of ulceration [33], 6mm slow-rebound cellular urethane and 6mm soft cellular urethane along the plantar contour (cushion-modified TCC) [34, 35], application of a felt deflective pad at the ulcer site [4] and ‘selective padding’ has been used to protect the toes, bony prominences and anterior lower leg [28]. Additionally, fibreglass materials are frequently used instead of Plaster of Paris [36–38] while conventional canvas cast shoes have replaced the rubber heel [39, 40]. The TCC has also been bi-valved to allow wound inspection and dressing, therefore rendering the cast removable [41, 42] however can be returned to a non-removable state with the use of a semi-rigid fibreglass bandage. Despite these modifications, the cast materials continue to be well moulded to the limb, with the materials closely approximating the contours of the limb ensuring the TCC is firm but not tight. Notwithstanding these modifications, the TCC acts by reducing localised pressure to the wound and immobilising the surrounding joints and soft tissue while preserving functional ambulation [1, 26, 29, 43]. Furthermore, the reduction of oedema [21, 29] and decreased intravascular fluid pressure improves microcirculation [44].

There are many factors contributing to the effectiveness of the TCC to offload pressure at the site of ulceration. Alteration of gait occurs with the TCC, such as a shorter stride length and velocity, thereby reducing the magnitude of pressure and cycles of repetition [26, 27, 43]. One major mechanism of action of the TCC has been attributed to “equalisation of plantar pressure”. This refers to the reduction and redistribution of weight-bearing pressure across the entire plantar surface of the foot, including those areas that do not normally bear a large load and by increasing the plantar surface contact area [1, 21, 26, 27, 29]. However, previous work indicated that this was not always the case. Contact area data and regional pressure patterns comparing a cushion-modified TCC and a conventional TCC have shown pressure reduction and redistribution without increasing the plantar contact area [34]. These findings support a suggested mechanism of load transfer to the walls of the TCC [1, 21, 26, 46] or to the rearfoot [26].

The walls of the TCC have been indirectly shown to bear approximately 30 % of the plantar load in small samples of healthy participants [21, 26, 45]. Direct measurement to measure load inside the TCC walls with capacitance sensors has shown that the TCC walls bear 23–34 % of the plantar load [46]. Furthermore, the direct measurement method has shown that the anterodistal and posterolateral-distal regions of the lower leg bear the highest load, although this has only been evaluated in two participants without an ulcer. Therefore, the objective of this study was to directly measure these two regions in patients with Diabetes and a plantar forefoot ulcer to further understand the mechanism of pressure reduction in the TCC.

## Methods

### Participants

Participants were recruited from the Foot Wound Clinic, Westmead Hospital, New South Wales, Australia. All participants had diabetes mellitus and plantar forefoot ulceration, and had provided informed written consent in accordance with the Human Research Ethics Committee (HREC 2009/12/5.12 (3093). Demographic details, medical history, physical characteristics and ulcer diagnostics were collected (Table 1).

**Table 1:** Demographics and physical characteristics of the sample (*n* = 17)

Variable	Total participants
Age (mean years, SD)	57.2 (12.9)
Gender, Male, no. (%)	14 (82.4 %)
Height (mean metres, SD)	1.76 (.06)
Weight (mean kg, SD)	102.5 (28.1)
BMI (mean kg/m^2^, SD)	33.6 (8.3)
Duration of Diabetes (mean years, SD)	18.1 (8.5)
Ulcer location	
Hallux, no. (%)	6 (35)
1^st^ MPJ, no. (%)	4 (23)
2^nd^ MPJ, no. (%)	1 (16)
3^rd^ MPJ, no. (%)	2 (12)
4^th^ MPJ, no. (%)	2 (12)
5^th^ MPJ, no. (%)	2 (12)
Ulcer duration (mean weeks, SD)	33.5 (61.9)
Toe pressure^a^ (mean PPG, SD)	104.1 (43.1)
Peripheral neuropathy^b^ (frequency, %)	17 (100 %)

*Note:* ^a^Toe pressure was assessed using photoplethysmography (PPG, Hadeco Smartdop 30 EX Vascular Ultrasound Doppler, Japan)

^b^Peripheral neuropathy was assessed with a neurothesiometer and 10g monofilament

### Intervention

**Fig. 1:**
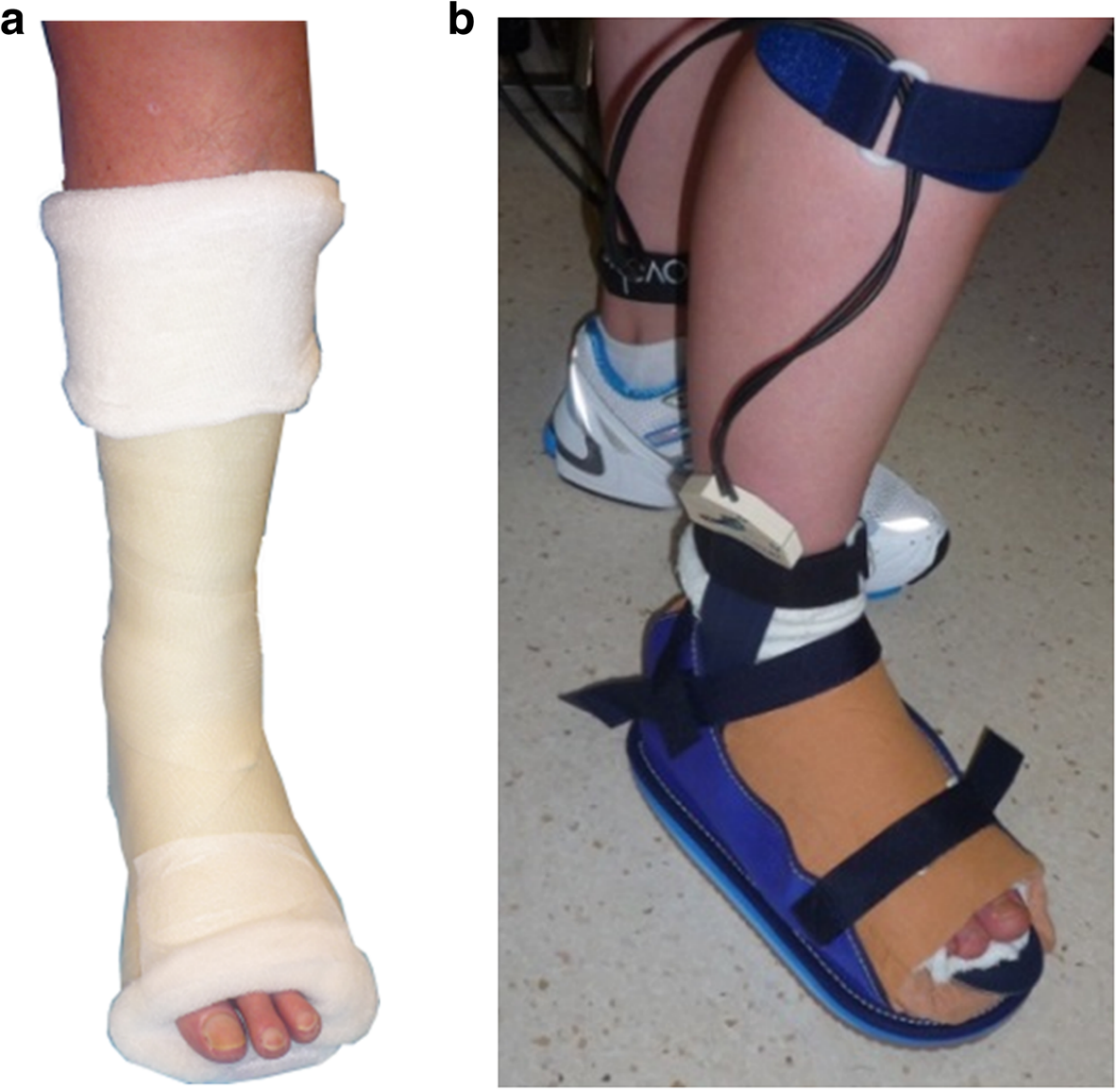
The TCC (**a**) and shoe-cast (**b**) conditions

An experienced podiatrist (LB) applied a cushion-modified TCC to each participant using a technique described previously [34]. Briefly, the technique involved a combination of rigid and semi-rigid cast materials, with the addition of a 6 mm slow-rebound cellular urethane and a 6 mm soft cellular urethane inlay (Fig. 1a). After 20 min, to allow for drying time as recommended by the manufacturer, each participant walked along a 9m walkway to familiarise themselves with walking whilst wearing the TCC. In order to accommodate the capacitance sensors, it was necessary to bi-valve the TCC. Returning the bi-valved TCC to a TCC was done with particular care using non-stretch tape to firmly reaffix the edges and align the cast walls to ensure the TCC integrity during data collection. To all intent and purposes, the TCC was considered to be as robust as a newly applied TCC. A standard canvas TCC shoe (Blue Denim Cast shoe, Secure, Taiwan) with a slight rocker-sole was used with the TCC to assist with propulsion. On the contralateral foot, a canvas TCC shoe with a 12mm cellular urethane inlay was worn to reduce any limb length difference between limbs. Following data collection, the walls of the TCC were removed to just below the malleolus to create a shoe-cast [21, 48] (Fig. 1b).

### Outcome measures

**Fig. 2:**
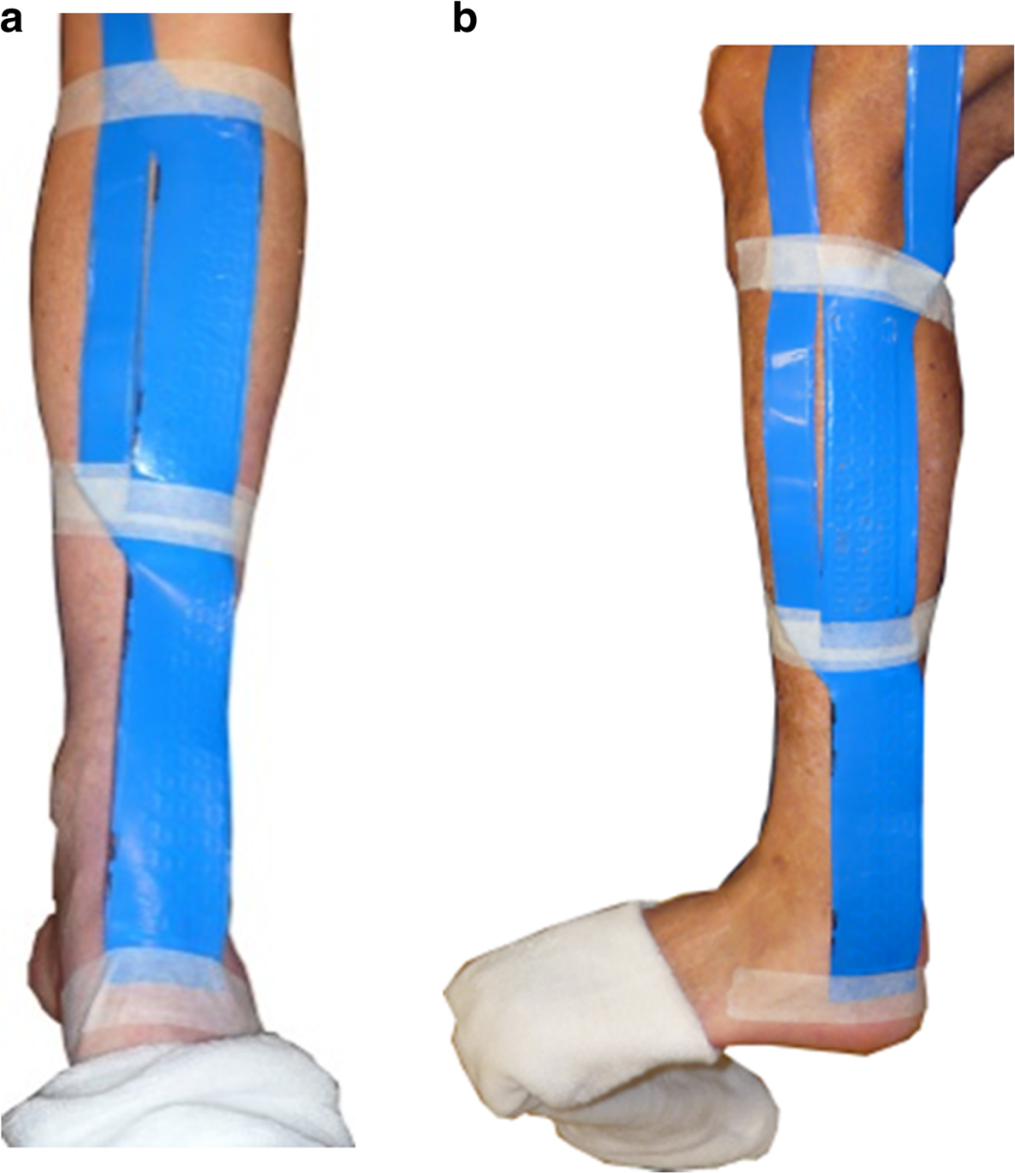
Capacitance sensor strips (pliance®, novel GmbH, Germany) placed at the anterodistal (**a**) and posterolateral-distal (**b**) regions of the lower leg

TCC wall load (contact area, peak pressure and max force) at two regions of the lower leg were evaluated with two capacitance sensor strips measuring 90 cm^2^ (pliance®, novel GmbH, Germany). These regions were based upon the proof of concept study identifying the areas of highest TCC wall load [46] and are defined as **anterodistal lower leg** (along the tibia, running distally, specifically across the top of the ankle mortise over connective tissue structures) and **posterolateral-distal lower leg** (on the posterolateral part of the lower leg, running distally from a line slightly posterior to the fibula head and passing posterior to the lateral malleolus, specifically at the area of the lateral malleolus) (Fig. 2). The pliance® sensor is < 1 mm thick and calibrated to a pressure range of 4.64-60kPa.

Plantar load (contact area, peak pressure and max force) was measured with a capacitance sensor insole with a resolution of 1.2 sensors per cm^2^ (pedar®, novel GmbH, Germany) placed between the plantar surface of foot (in stockinet) and the cellular urethane material inside the TCC. Pedar® is an accurate, reliable, and valid system [47, 48].

### Experimental protocol and data analysis

The participants walked at a comfortable self-selected walking speed along a 9m walkway. Trials, whereby the participants walked at a velocity outside a 10 % individual tolerance, were excluded from the study. Both pedar® and pliance® collected data simultaneously at a sampling rate of 50Hz synchronised to the temporal event of heel strike. Each trial started with the participant standing with two feet together. Each participant was instructed to commence walking after the various data collection systems had commenced recording. In this way, the heel strike of the first step of each trial could be used for synchronisation purposes. This synchronisation was completed manually by combining video, plantar and TCC wall data. Each participant walked a minimum of two successful trials.

Descriptive statistics and normality of data distribution were computed in SPSS v22.0 (IBM SPSS Statistics for Windows, Armonk, NY, USA). The magnitude of TCC wall load as a proportion of plantar load was calculated. Following removal of the cast walls, the difference in plantar loading between the TCC and the shoe-cast for the whole foot and three clinically relevant regions of the foot, including rearfoot, midfoot and forefoot (region of interest as the site of ulceration), were calculated using the Novel software (novel GmbH, Germany) [49]. Paired sample t-tests were undertaken to assess significance between TCC and shoe-cast conditions. The alpha value was set at 0.05.

## Results

Seventeen patients with diabetes and a forefoot ulcer volunteered to participate in this study. Walking speed was comparable between conditions (TCC 0.77 +/- 0.21 m/s, shoe-cast 0.79 +/- 0.23m/s) (*p* > 0.05).

Contact area, max force and peak pressure inside the walls of the TCC at the anterodistal and posterolateral distal regions of the lower leg are shown in Table 2.

**Table 2:** Descriptive *novel pliance*® data for the walls of the TCC (*n* = 17)

Region of interest	Contact area [cm^2^]	Peak pressure [kPa]	Max force [N]
Antero-distal lower leg	71.0 (12.2)	44.1 (15.2)	74.9 (35.2)
Postero-lateral distal lower leg	51.2 (13.3)	18.4 (15.2)	24.7 (26.5)

*Note:* Data are mean (SD)

The anterodistal lower leg recorded 48 % and the posterolateral distal lower leg recorded 34 % of the *mean plantar contact area* of the whole foot in the TCC.

The anterodistal lower leg recorded 28 % and the posterolateral distal lower leg recorded 12 % of the *mean plantar peak pressure* of the whole foot in the TCC.

The anterodistal lower leg recorded 12 % and the posterolateral distal lower leg recorded 4 % of the *mean plantar max force* of the whole foot in the TCC.

Differences in contact area, max force and peak pressure between the total contact cast and the shoe-cast for the whole foot, rearfoot, midfoot and forefoot (region of ulcer) are shown in Table 3. When the TCC was cut down to a shoe-cast, contact area significantly increased by 7.1 cm^2^ (5 %) beneath the whole foot, 4.3 cm^2^ (8 %) at the midfoot and 4.7 cm^2^ (6 %) at the forefoot (*p* < 0.05). Peak pressure significantly increased by 8.9 kPa (8 %) beneath the midfoot and by 17.5 kPa (13 %) at the forefoot (*p* < 0.05). Max force significantly increased by 13.2 N (6 %) beneath the midfoot (*p* < 0.05).

**Table 3:** Plantar pressure data for the total contact cast compared to the shoe-cast (*n* = 17)

Region of interest	Contact area [cm^2^]	Peak pressure [kPa]	Max force [N]
Total Contact Cast			
- Whole foot	149.6 (18.1)	158.8 (52.6)	592.4 (201.1)
- Rearfoot	19.7 (5.5)	128.5 (45.0)	130.0 (59.4)
- Midfoot	54.5 (6.2)	112.9 (42.3)	213.5 (85.6)
- Forefoot	75.5 (12.1)	131.4 (59.5)	247.8 (122.2)
Shoe-cast			
- Whole foot	156.7 (11.9)*	171.1 (57.5)	602.1 (176.9)
- Rearfoot	19.7 (5.6)	131.6 (43.1)	106.7 (66.2)
- Midfoot	56.8 (4.7)*	121.8 (37.2)*	226.7 (83.3)*
- Forefoot	80.2 (8.8)*	148.9 (64.5)*	268.7 (116.5)

*Note:* Data are mean (SD)

*Significant difference compared to the total contact cast (*p* < 0.05; *paired* samples t*-*test)

## Discussion

### Main findings

In patients with diabetes and a forefoot plantar ulcer, the walls of the TCC recorded considerable load (contact area, peak pressure and max force). It is of interest that the region of greatest load (anterodistal lower leg), did not occur at the rigid cast walls but at a region whereby the limb was encased with Soft Cast™ which is a semi-rigid cast material. This finding supports the literature that diligent moulding of the cast material around body contours can increase the level of stiffness and strength of the cast material to carry substantial load [52].

There were significant differences in plantar load (contact area, peak pressure and max force) between the TCC and the cut down shoe-cast for the whole foot, rearfoot, midfoot and forefoot (region of ulcer). An interesting finding was that there was a significant decrease of the plantar contact area in the TCC compared to the shoe-cast, with concomitant lowering of plantar peak pressure beneath the midfoot and forefoot in the TCC. These results contradict some aspects of the “equalisation of plantar pressure” theory [1, 21, 26, 29]. Instead, the reduced plantar contact area in the TCC, compared to the shoe-cast, suggests that the foot is suspended by the considerable load bearing capacity of the walls of the TCC, which contributes mechanically to the pressure reduction and redistribution properties of the TCC. Our data support the load transference theory of the TCC [21, 26, 45] which we can now measure directly with capacitance sensors.

### Comparison with the literature

Various studies have assessed load transference to the cast walls by indirect methods in small samples of healthy participants. Shaw and colleagues [26], simultaneously collected pedar® data beneath the foot of five participants with elevated forefoot pressures wearing a TCC (modified with forefoot sifoam), whilst walking across a force platform. It was reported that the difference in impulse, with the plantar insole reporting smaller values than the force platform, was indicative of load transfer to the cast wall. This difference was calculated as 31.2 % of the impulse measured by the force plate. Leibner and co-workers collected pedar® data beneath the foot in 12 healthy participants wearing a TCC during walking. The TCC was then cut-down to produce a shoe-cast and data collection was repeated [21]. The smaller values for average force per step in the TCC condition were attributed to a transfer of load to the cast walls compared to the shoe-cast condition. This transfer of load was calculated to be 36 % of the average force per step measured in the shoe-cast condition. Finally, Tanaka and colleaugues [45], assessed five healthy participants and measured plantar pressure (F-SCAN, Tekscan Inc, South Boston, Massachusetts) in a conventional Patella Tendon Bearing Cast (PTB) with a heel (used for the treatment of below knee fractures), and the contralateral side (extension shoe) and attributed a 30 % offloading to the cast walls. Direct measurement of inside the TCC walls with the use of capacitance sensors has shown that the TCC walls bear 23–34 % of the plantar load [46]. It is diffcult to compare these studies because they all report different units of measurement, cast technique, terminal devices (cast shoes and heels), TCC rigidity and cushioning materials. Neverthless, the reduction of plantar load via transfer to cast walls seems to be in the vicinity of 30 %.

### Clinical implications

The mechanism of action, that suspension of the foot by the considerable load bearing capacity of the TCC walls contributes to the pressure reduction and redistribution properties of the TCC, is supported by the literature [21, 26, 34, 45]. It is known that sufficient tissue is required at the plantar metatarsal area to allow soft tissue compression under load bearing for pressure distribution [51] and that there is a strong inverse relationship between plantar tissue thickness and plantar pressure magnitude in patients with diabetes and neuropathy [52]. In this study, the TCC was cushion-modified by incorporating a full-length 6mm slow-rebound cellular urethane and 6mm soft cellular urethane inlay, as result of previous data demonstrating superior offloading compared to a conventional TCC [34]. It is postulated that the 12mm cellular urethane acts to enhance the plantar fat pad thickness, improve the shock absorption capabilities and to act as a buffer to create a cavity between the plantar surface of the foot and the rigid and semi-rigid cast material. This concept is supported by Tanaka *et al*. who found that the addition of a 10mm soft sponge in a conventional Patella Tendon Bearing Cast (PTB) cast increased offloading from 30 % to 56 % by producing a space between the foot and the cast. Furthermore, when a 30mm space was created via an air bellows bag, there was a 100 % offloading. Tanaka *et al*. suggested that this space results in suspension of the leg by the inner region of the cast [45]. Shaw *et al*. refer to the contribution of the soft foam beneath the forefoot as creating a cavity and removing the load bearing surface from the metatarsal heads to suspend the foot [26].

Based on this study and previous studies in the literature, the offloading mechanism of the TCC can be attributed to a combination of the following factors:

- Suspension of the foot by the considerable load bearing capacity of the TCC walls, especially the anterodistal and the posterolateral-distal regions of the lower leg.
- Reduction of plantar pressure by incorporating a full-length 6mm slow-rebound cellular urethane and 6mm soft cellular urethane inlay inside the TCC.
- Redistribution of plantar pressure by a well-moulded TCC using a combination of rigid and semi-rigid cast materials, extending proximal to the ankle.
- Fixation of the ankle at 90^0^ within the TCC, thereby eliminating plantarflexion and dorsiflexion. The addition of a cast shoe with a rockersole assists with propulsion.
- Forced compliance and alteration of gait, such as shorter stride length and velocity.

Therefore in addition to biomechanical factors in reducing plantar forefoot load, wearing a TCC alters the patient behaviour in terms of “forced” compliance and acting to reduce activity levels [25]. There is a reduction of the number of steps per day and therefore a concomitant reduction of cumulative plantar load and stress [53]. Thus the combination of biomechanical factors and alteration of behaviour contributes to the successful wound healing attributes of the TCC.

The results of this study also suggests that when prefabricated devices are selected to offload forefoot plantar ulceration, that the design of such device should mimic a TCC and incorporate rigid “walls” extending proximally, medially and laterally at the lower leg, with the lower limb fixed securely to minimise shear forces and should contain a cushioning insole and be fixated at the ankle at 90° with a rockersole.

Additionally, whenever possible prefabricated devices should be made non-removable using a semi-rigid cast bandage or cohesive bandage. Furthermore, that the device should be lightweight and be of low profile to encourage adherence and minimise apparent leg length discrepancy. If oedema exists, compression should be applied. It is recommended that the patient be advised to take shorter, slower steps and restrict activity levels. All these factors will contribute to enhance ulcer healing.

### Limitations

Due to technical limitations, only perpendicular forces were measured and the cast wall load area of measurement was limited to 90cm^2^. Notwithstanding, in regard to the cast wall data, this is the first study to measure such load directly in a patient population. A further limitation of this study is that it was cross-sectional and that TCC wall load characteristics over time were not assessed. The majority of participants in this study were representative of patients presenting with plantar forefoot ulceration to our tertiary hospital. As such, while the results may have differed in people with lower BMI, the findings would not have been generalisable to the relevant clinical population.

## Conclusion

Offloading plantar neuropathic ulcers using a TCC is regarded as the ‘gold standard’ in patients with diabetes. This study shows that the walls of the TCC bear considerable load when measured directly in patients with diabetes and forefoot ulceration. Reduced plantar contact area in the TCC, compared to the shoe-cast, suggests that the foot is suspended by the considerable load bearing capacity of the walls of the TCC, which supports the load transference theory and contributes mechanically to the pressure reduction and redistribution properties of the TCC. Limb suspension seems to be an important component of effective TCC offloading, particularly when used in conjunction with cushioning materials. Any off-the-shelf offloading device should mimic these essential design features of the TCC.
